# Predictive multi-omic biomarkers for urban zoonotic spillover detection: an integrative review

**DOI:** 10.3389/fpubh.2025.1720300

**Published:** 2026-01-14

**Authors:** Iliana C. Martínez-Ortiz, Igor Garcia-Atutxa, Javier I. Sanchez-Villamil, Carlos Machain-Williams, Miguel Angel Reyes-López, Francisca Villanueva-Flores

**Affiliations:** 1 Centro de Investigación en Ciencia Aplicada y Tecnología Avanzada (CICATA) Unidad Morelos del Instituto Politécnico Nacional (IPN), Xochitepec, Morelos, Mexico; 2 Escuela Politécnica Superior, Universidad Católica de Murcia (UCAM), Murcia, Spain; 3 Estudios en Una Salud, Unidad Profesional Interdisciplinaria de Ingeniería Campus Palenque, Instituto Politécnico Nacional, Palenque, Chiapas, Mexico; 4 Centro de Biotecnología Genómica, Laboratory of Conservation Medicine, Instituto Politécnico Nacional, Reynosa, Tamaulipas, Mexico

**Keywords:** multi-omics surveillance, urban wildlife reservoirs, zoonotic spillover detection, one health approach, predictive biomarkers

## Abstract

Urban wildlife is an overlooked yet critical component of zoonotic disease surveillance, especially in biodiversity hotspots where human–animal interfaces accelerate spillover risk. This review synthesizes five complementary omics layers: Host microRNAs, host–pathogen genetic markers, bacterial microbiome profiling, viromics, and host transcriptomics into a single predictive framework for early spillover detection. Across taxa and pathogen classes, we highlight convergent molecular signatures of infection, from receptor polymorphisms and shifts in MHC diversity to pathogen-responsive miRNAs, high-risk bacterial genera, novel viral sequences, and transcriptomic profiles associated with pathogen tolerance. By integrating these biomarkers into a cross-validated, multi-omics architecture, we outline a workflow from non-invasive sampling to predictive modeling that enhances sensitivity for detecting both known and cryptic pathogens. We also identify key barriers, including Field preservation, cross-species assay standardization, and bioinformatics capacity, and propose practical solutions, such as interoperable pipelines and open-access databases. This integrative approach shifts surveillance from reactive detection to anticipatory risk profiling, providing a transformative tool for One Health strategies aimed at forecasting and preventing zoonotic epidemics.

## Graphical Abstract

**Figure:**
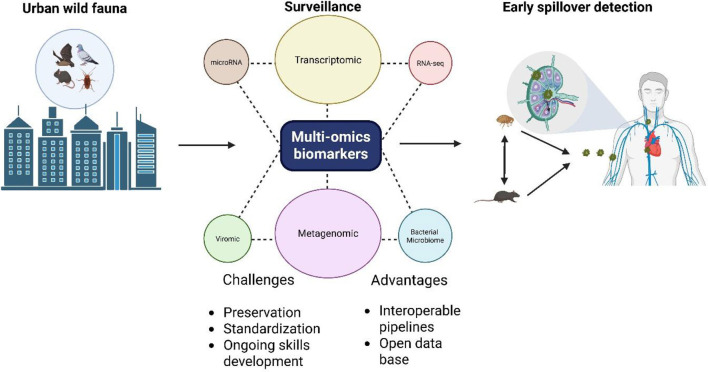

## Introduction

Zoonotic spillovers represent an increasing global health threat. Nearly 60% of all human infectious diseases are zoonotic in origin, and at least 75% of new emerging infectious diseases arise from animal reservoirs (1). Environmental and anthropogenic changes, from climate shifts to wildlife–human encroachment, are accelerating the discovery of novel pathogens in animals (2). The COVID-19 pandemic, traced to a wildlife origin, exemplifies how quickly an animal virus can ignite a global crisis (3). The scale of the threat is immense: Mammals alone are estimated to harbor a minimum of ~320,000 undiscovered viruses, capable of spilling over. However, conventional wildlife surveillance, however, struggles to detect these looming pathogens. Field surveys for viruses face

logistical and technical hurdles; many viruses circulate at very low prevalence in wild populations, requiring enormous sample sizes for detection (4). Traditional monitoring often targets a short list of known pathogens. It relies on symptomatic or dead animals, thereby missing asymptomatic carriers (which are common in reservoir hosts) and entirely novel agents (1). In short, our current surveillance paradigm is mainly reactive and biased toward viruses already implicated in outbreaks (5). This leaves a vast “hidden virome” in wildlife unchecked, underscoring the urgent need for more sensitive and predictive surveillance approaches.

To overcome these limitations, researchers are adopting integrated multi-omics frameworks that capture multiple molecular signals of infection. By combining diverse data layers (genomic, transcriptomic and beyond), multi-omics surveillance can detect subtle host–pathogen interactions that conventional single-marker tests ofeten overlook (5). Each omics technique contributes unique insights into early pathogen detection and disease dynamics.

MicroRNA (miRNA) profiling is an emerging method in this context. Circulating miRNAs (small regulatory RNAs of ~22 nucleotides), are released during the host's early immune response and serve as stable biomarkers, even before direct pathogen detection or seroconversion is possible. Notably, distinct host miRNA signatures have been documented in initial infection stages of severe zoonotic pathogens such as Hendra virus and Ebola (6, 7).

Similarly, microbiome sequencing via 16S rRNA gene analysis offers a powerful surveillance tool. Because pathogen invasion frequently disrupts the host's commensal microbial communities, significant shifts in microbiota composition can signal early disease onset. High-throughput 16S rRNA sequencing in vectors and wildlife populations has demonstrated remarkable sensitivity in detecting emerging zoonotic bacteria, providing broad-spectrum coverage and enabling early identification of pathogenic threats within animal communities (8). A host's microbiome thus serves a meaningful early indicator of wildlife health and infection status (9).

Viromics, or unbiased metagenomic sequencing of viruses, further strengthens zoonotic surveillance. By analyzing environmental samples or host tissues without preconceived assumptions, researchers can directly identify previously unknown viruses circulating in wildlife reservoirs. Viral metagenomic studies, particularly in wild birds and other reservoir species, have revealed diverse and previously unrecognized viral communities, effectively establishing an early-warning system for potentially zoonotic viral pathogens (10).

Lastly, host–pathogen transcriptomics approaches, such as dual RNA-Seq, simultaneously profile RNA from both the host and the pathogen within infected tissues. This strategy uncovers reciprocal molecular dynamics, detailing how host gene expression, including immune and stress responses, is modulated upon pathogen invasion, while concurrently characterizing pathogen-specific gene activities. Such dual transcriptomic analyses have revealed hidden molecular interactions, including pathogen virulence factors and regulatory non-coding RNAs, which single-sided assays frequently miss. Comprehensive host–pathogen gene interaction data obtained from these studies are instrumental for elucidating mechanisms of pathogen adaptation, spillover and ultimately disease emergence (11).

By integrating these omics layers into a unified surveillance platform, we gain a comprehensive view of infection that transcends the limits of any single method. An early signal (be it a telltale microRNA, a bacterial dysbiosis, or a snippet of viral RNA) can be cross-validated and contextualized with other omics evidence, markedly enhancing confidence in detection. Equally important, the multi-omics approach enriches our understanding of pathogen ecology and host responses, from immune gene upregulation to microbiome disruptions, thereby improving our ability to predict which animal infections are poised to jump between species. Studies leveraging multi-omics and host–pathogen interaction data have already provided new insights into the genetic diversity and transmission dynamics of zoonotic agents. They highlight the need for large-scale, interdisciplinary surveillance grounded in a One Health perspective (1). In essence, an integrated multi-omics framework offers a proactive strategy to detect cryptic zoonotic threats, decode their emergent behavior, and enable data-driven interventions before isolated spillover events escalate into pandemics.

In light of these challenges, the present review focuses on identifying and mapping the key molecular markers captured by emerging multi-omics techniques, as well as demonstrating how this integrated approach can strengthen early zoonotic disease surveillance. Specifically, we highlight critical infection signals, from host microRNA signatures and microbiome dysbiosis to novel viral sequences and host–pathogen transcriptional shifts, as actionable biomarkers detectable by these multi-omic approaches at the wildlife–human interface. This focus addresses a crucial gap in the current literature: While individual omics studies have revealed various early-warning signals of infection, no comprehensive synthesis has yet contextualized these disparate biomarkers within a unified One Health surveillance framework (1). By consolidating these multi-layered molecular indicators and examining their synergistic potential, our review provides a timely resource to guide more proactive and predictive monitoring strategies for zoonotic spillovers. Ultimately, this integrated multi-omics perspective is poised to enhance our ability to identify cryptic threats and inform interventions well before incipient spillover events can escalate into public health crises.

**Figure 1:**
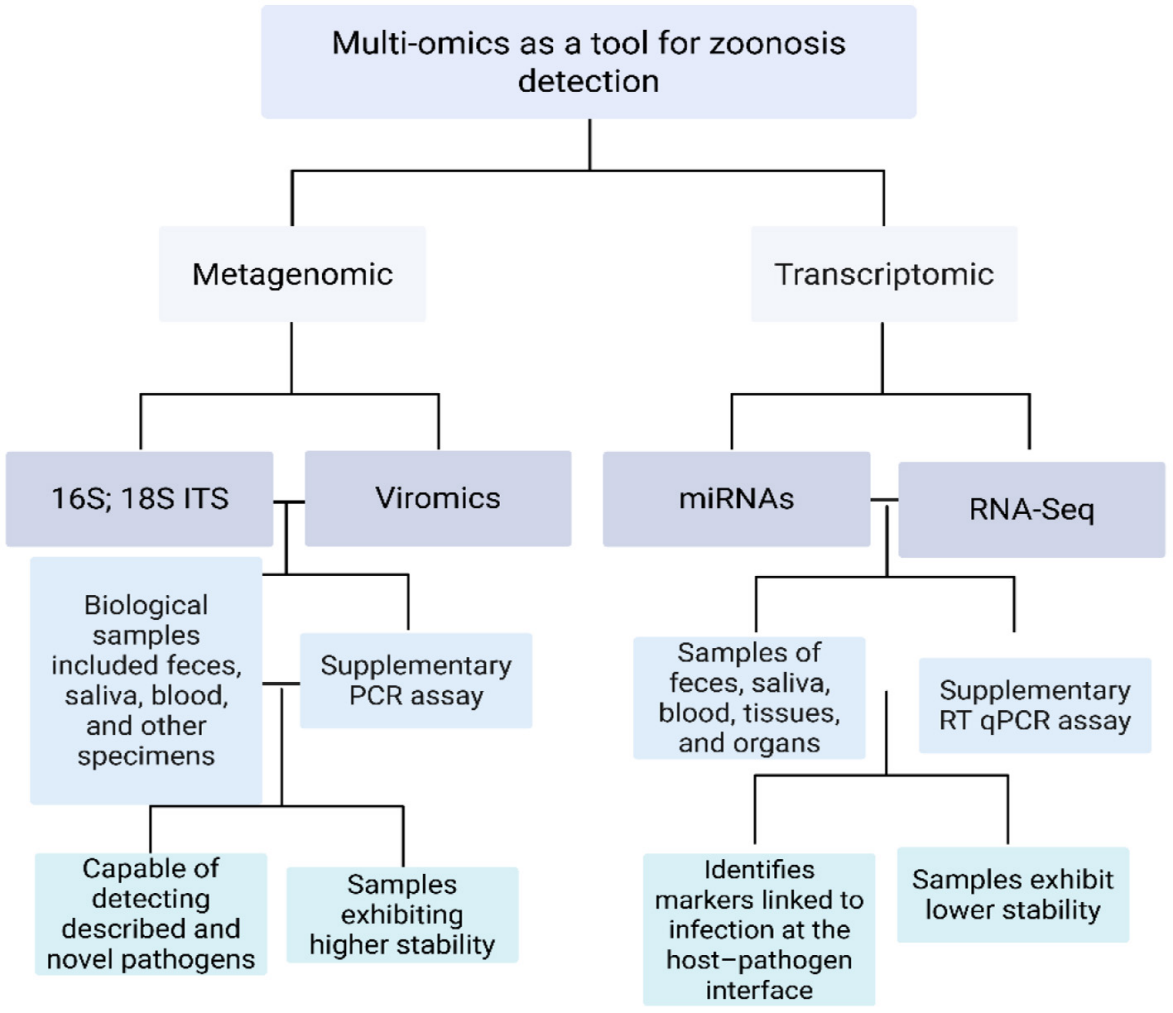
Multi-omics workflow for urban zoonotic-spillover surveillance. The metagenomic branch (16S/18S–ITS profiling and viromics) and the transcriptomic branch (miRNA panels and RNA-seq) are shown with typical non-invasive sample types (feces, saliva, blood, tissues), preservation constraints (higher nucleic-acid stability vs. lower RNA stability), and complementary confirmation assays (PCR/RT-qPCR). Together, these layers enable detection of both known and novel pathogens and the identification of host–pathogen biomarkers for early risk profiling at the wildlife–human interface. ITS: internal transcribed spacer; RT-qPCR: reverse-transcription quantitative PCR.

Figure 1 presents an integrative overview of the multi-omics surveillance workflow for urban zoonotic spillover: it traces the path from non-invasive sampling and field preservation quality to complementary assays (host miRNAs, host–pathogen genetic markers, 16S rRNA microbiome profiling, viromics, and host/dual RNA-seq) and their integration into a cross-validated, predictive model for early risk profiling at the wildlife–human interface.

## Methodology

This review was designed as a narrative, integrative synthesis of peer-reviewed literature addressing the application of multi-omics approaches to the surveillance of zoonotic pathogens in urban wildlife. The purpose was not to produce a systematic review but to integrate evidence across diverse omics domains into a coherent framework, while maintaining methodological transparency to strengthen reproducibility and credibility.

Relevant studies were identified through a targeted search conducted between January and March 2025 in Web of Science, PubMed, Scopus, and Google Scholar, supplemented by reference mining from key papers. Search terms combined descriptors for molecular biomarkers (“microRNA,” “miRNA,” “genetic polymorphism,” “immune gene”); omics approaches (“microbiome,” “metagenomics,” “viromics,” “viral metagenomics,” “transcriptomics,” “RNA-seq”); wildlife ecology (“urban wildlife,” “synanthropic species,” “rodents,” “bats,” “pigeons,” “urban birds”) and zoonotic diseases (“zoonotic pathogens,” “spillover,” “One Health”). The temporal scope was restricted to publications in English from January 2000 to February 2025, corresponding to the period during which modern omics technologies became established in wildlife pathogen surveillance.

Inclusion criteria required that studies: (1) Reported original molecular or omics data from wildlife species inhabiting urban or peri-urban environments; (2) Provided evidence of association with known or potential zoonotic pathogens and 3) Applied at least one of the five omics layers considered in this review: Host microRNAs, host–pathogen genetic markers, 16S rRNA microbiome profiling, viral metagenomics, or host transcriptomics.

Exclusion criteria applied to studies that: (1) Focused solely on domestic animals without any wildlife interface; (2) Lacked molecular or omics data (e.g., ecological observations without laboratory analysis), and (3) Were limited to *in vitro* or experimental infections without field relevance, unless these provided essential insights for interpreting biomarkers in wildlife contexts.

From each study, we recorded host species, ecological setting, pathogen identity, zoonotic relevance, omics methodology, and identified biomarkers. Quantitative measures (e.g., prevalence, fold change, binding affinity) were included only when reported by the original authors, used solely to illustrate trends, and were neither pooled nor statistically synthesized. Findings were interpreted within broader ecological and epidemiological contexts to support cross-omic integration. Although exhaustive coverage was not intended, selection bias was minimized through multi-database searches, inclusion of diverse geographic regions, and predefined criteria emphasizing methodological rigor and relevance to urban wildlife spillover. This synthesis underscores the value of each omics approach and its potential for cross-validation, thereby informing predictive One Health surveillance.

## MicroRNAs (miRNAs) are emerging as informative surveillance biomarkers in urban wildlife reservoirs (rodents, bats, birds) carrying zoonotic viruses, bacteria, and parasites

These ~22-nt regulatory RNAs orchestrate host immune responses and can be sampled via minimally invasive methods (e.g., blood draws or swabs), offering a window into subclinical infections. Numerous studies document pathogen-specific miRNA expression shifts; for instance, foot-and-mouth disease virus in cattle induces distinct serum miRNA patterns, with miR-17-5p surging during acute infection and miR-31 highest in persistent carrier phases (12). Parasitic infections likewise perturb host miRNA profiles. *Echinococcus* (cestode) infection in mice altered 58 circulating miRNAs (21 upregulated, 37 downregulated) by 4 weeks post-infection, including pro-inflammatory miR-146a-5p and miR-21a-3p, which rose significantly at later stages. Remarkably, in Ebola virus disease (a bat-borne zoonosis), an Ebola-encoded miRNA-like fragment (miR-VP-3p) becomes detectable in patient serum before viral RNA, and an 8-miRNA host classifier could correctly identify 86% of infection cases (64/74), even flagging 50% of presymptomatic individuals (12). By contrast, bacterial zoonoses (e.g., leptospirosis in rats) remain less well-characterized in terms of miRNA biomarkers; it is anticipated that persistent bacterial infections modulate host miRNAs similarly, but such responses in wild reservoirs have yet to be systematically profiled (13, 14). These findings highlight that miRNA signatures span viral, parasitic, and bacterial infections, underscoring their potential as broad-spectrum indicators of zoonotic risk (15–17). Importantly, miRNA readouts are mechanistically linked to pathogen biology. Some pathogens even release their miRNAs (or miRNA-mimics) that circulate in the host, as seen with certain viruses and helminths (17, 18), adding another layer of detectable signals for early diagnosis of infections.

Circulating miRNAs are remarkably stable in biofluids and survive harsh conditions, facilitating field sampling. For example, miRNA profiles from sera stored for 10–15 years at −20 °C remain comparable to those from fresh samples, and plasma miRNAs persist with minimal degradation for ~1 h at ambient temperature (although significant loss occurs by 24 h) (12). This inherent stability permits the collection of blood or saliva from urban animals *in situ* and its transport to the lab for analysis. The gold standard for miRNA quantification is reverse-transcription quantitative PCR (RT-qPCR), using either stem-loop primers or poly(A)-tailing plus custom primers (19). RT-qPCR assays achieve high sensitivity (down to femtomolar levels) but require carefully normalized and typically target known miRNAs. For broader screening, hybridization arrays and digital counting platforms are available. Notably, the NanoString nCounter system can quantify ~800 miRNAs simultaneously without PCR amplification, offering high throughput and sensitivity comparable to PCR in a single assay (20). Small RNA sequencing (miRNA-seq) is another pivotal tool; it profiles the entire miRNA repertoire of a sample *de novo*, which is crucial when working with wildlife species lacking complete miRNA annotations. Unlike targeted assays, miRNA-seq can discover novel miRNAs indigenous to a host species or pathogen. However, sequencing data requires careful interpretation, as platform-specific biases and “noise” reads can occur (21). In practice, a typical workflow might involve using miRNA-seq on a subset of samples from, say, urban bats to identify candidate biomarkers, then developing targeted RT-qPCR or NanoString panels to monitor those miRNAs across larger wild cohorts. The choice of platform also depends on field logistics: Portable PCR machines and cartridge-based miRNA tests are being explored for point-of-care wildlife diagnostics, whereas high-throughput sequencing is usually confined to laboratory settings for in-depth discovery. Regardless of the method, even small volumes of serum or plasma (≪1 ml) from a captured animal are sufficient for robust miRNA profiling, highlighting the feasibility of miRNA-based zoonotic surveillance in practice (22).

One primary consideration is data normalization to account for variable RNA yields and experimental noise. Appropriate normalization strategies include the use of exogenous spike-in controls and, critically, endogenous reference miRNAs that are stably expressed. Recent studies have defined panels of invariant miRNAs for normalization in plasma RT-qPCR assays, and guidelines recommend incorporating multiple reference miRNAs or small RNAs to improve quantification reliability (19). Quality control is also essential: For example, hemolysis during sample handling can release abundant erythrocyte miR-451 and other cellular miRNAs that skew profiles, thus a high miR-451/miR-23a ratio in a sample serves as a red flag for blood cell contamination (23). Technical caveats unique to wildlife studies include cross-species differences in miRNA sequences. Even conserved miRNAs may have single-nucleotide variants in, say, bat or bird genomes that necessitate species-specific primers or probes. The limited genomic resources for many wildlife species mean that profiling their miRNAs often starts with a discovery phase (RNA-seq) to identify the miRNA catalog (13, 14, 24). Moreover, distinguishing pathogen-induced miRNA signatures from other environmental or physiological factors requires careful experimental design. Wild animals often experience stress, nutritional variation, and co-infections, all of which can influence miRNA levels. Longitudinal sampling and appropriate uninfected controls (or baseline data from captivity) can help attribute observed miRNA changes to a specific pathogen exposure rather than confounders. Another challenge is maintaining RNA integrity from field to lab: Although miRNAs are more stable than mRNAs, prompt sample processing or the use of RNA stabilizing reagents is advised, since even hardy miRNAs show degradation after a day at room temperature (25). Finally, integrating miRNA biomarkers into wildlife disease surveillance must take into account both performance and practicality. Assay sensitivity and specificity need validation against gold-standard pathogen tests. Encouragingly, some miRNA panels have already demonstrated high accuracy (e.g., >85% in Ebola infection classification) (15) and the ability to detect infections earlier than PCR (6). On the practical side, current miRNA profiling can be resource-intensive (costlier than routine PCR or ELISA) and demands molecular biology expertise, which may limit immediate field adoption. Nevertheless, as techniques become more affordable and automated, miRNA assays could represent a transformative addition to One Health surveillance, enabling non-lethal, broad-spectrum monitoring of urban wildlife for signs of zoonotic pathogen circulation. In conclusion, miRNA biomarkers constitute a precise and biologically grounded tool, from virus-reactive miR-146a or miR-181 indicating immune activation (26) to parasite-derived miRNAs signaling occult infections (27), but realizing their full potential in urban wildlife will require meticulous methodological standards and cross-validation to ensure robust, actionable insights.

Table 1 summarizes key miRNAs reported in diverse urban wildlife species (including rodents, bats, birds, and carnivores), detailing their relevance as biomarkers for various zoonotic viruses, bacteria, and parasites. The table highlights both experimentally validated and computationally predicted miRNAs, specifying their expression patterns, quantitative metrics, and the sample types analyzed. Notably, miRNAs such as miR-181 and miR-155 display consistent upregulation during viral and bacterial infections, respectively, while parasite-derived miRNAs like miR-71 serve as precise indicators of parasitic infestations. This integrative overview underscores the broad applicability of miRNAs as minimally invasive and robust surveillance tools, capable of detecting zoonotic risks in urban ecosystems, thereby facilitating timely interventions and disease prevention efforts.

**Table 1:** Emerging miRNA biomarkers in urban wildlife reservoirs of zoonotic diseases.

**miRNA biomarker**	**Origin**	**Animal reservoir (host)**	**Zoonotic pathogen/ disease**	**Evidence and key findings**	**References**
miR-181 family (e.g., miR-181a/b)	Host miRNA	Fruit bats (spillover to horses, etc.)	Hendra virus (*Henipavirus*), viral encephalitis	Validated findings indicate that immune-responsive miR-181 is significantly elevated in body fluids during Hendra virus infection. In infected animals (ferrets, horses), higher miR-181 levels correlate with increased virus-induced cell–cell fusion, suggesting its potential as an early infection marker. Additionally, pro-viral roles for miR-17~92 family members have been identified, supporting host miRNAs as surveillance targets in henipavirus outbreaks.	(26)
miR-8066, miR-5197, miR-3611, miR-3934-3p, miR-1307-3p, miR-3691-3p, miR-1468-5p	Viral and host (conserved)	Bats, pangolins, and civets *(predicted carriers)*	SARS-CoV-2 (COVID-19 coronavirus)	Predicted viral microRNA-like sequences from SARS-CoV-2 resemble host miRNAs. For example, miR-1307-3p and miR-5197 are conserved among the virus and multiple mammals (bat, pangolin, pig). These viral miRNA mimics could modulate host immunity and are being explored as biomarkers for COVID-19 spillover (*in silico* evidence).	(81)
Multiple miRNAs (163 identified, incl. novel bird-specific miRNAs)	Host miRNAs	Wild birds (shorebirds on urban coasts)	Avian influenza (low-pathogenic AIV)	Validated profiling in wild ruddy turnstones identified 163 miRNAs (including two novel avian miRNAs) in blood. Levels of specific miRNAs shifted in birds infected with low-pathogenic avian flu compared to uninfected birds. Differences in particular miRNAs correlated with age, sex and infection status Suggesting potential miRNA signatures for monitoring flu exposure in urban bird populations (e.g., abundant liver-associated gga-miR-122).	(82)
miR-28-5p, miR-302c-3p, miR-302a-3p	Host miRNAs	Rodents (urban rats/mice)—*studied in humans*	*Leptospira*. (Leptospirosis bacterial infection)	Validated: miR-28-5p, miR-302c-3p and miR-302a-3p were significantly upregulated during acute human leptospirosis (proxy for rodent-borne infection), correlating with immune response pathways. These miRNAs are proposed as early diagnostic biomarkers and potential surveillance markers for leptospiral carriage in urban rodents, pending validation in reservoir hosts.	
miR-155-5p	Host miRNA	Wild ungulates (e.g., urban deer), cattle *(spillover model)*	*Mycobacterium bovis* (zoonotic TB in wildlife)	Validated: miR-155, a known immune-responsive miRNA, is significantly elevated during tuberculosis infection. In cattle infected with *M. bovis*, higher miR-155 levels correlated with increased disease severity, suggesting its utility as a biomarker for infection and progression. Monitoring miR-155 in blood or milk may differentiate infected from vaccinated animals, aiding wildlife TB surveillance (e.g., deer serum testing in urban-adjacent populations).	(83, 84)
cfa-miR-346 (canine miR-346)	Host miRNA	Stray dogs (urban reservoir of leishmaniasis)	*Leishmania infantum* (Visceral leishmaniasis protozoan)	Validated: miR-346 is upregulated in human and canine macrophages infected with *L. infantum*. Plasma miR-346 is higher in naturally infected dogs than in uninfected ones, suggesting it as a minimally invasive marker for canine *Leishmania* infection. Despite individual variability, elevated miR-346 could help detect infections in urban dog populations before clinical signs appear.	(85)
Parasite miR-71 (and co-expressed “bantam” and let-7 variants)	Parasite miRNA	Various urban wildlife (canids, rodents) that host helminths	Helminth parasites (e.g., *Schistosoma, Echinococcus*)	Validated: Parasite-secreted miR-71 and let-7 miRNAs are reliable cross-species helminth infection biomarkers. *Schistosoma japonicum* releases sja-miR-71 and sja-let-7, detectable in host blood. Similarly, egr-miR-71 and egr-let-7 from *Echinococcus tapeworms* appeared consistently in plasma from hydatid patients but not controls, decreasing after cyst removal. Thus, detecting parasite miR-71/let-7 in blood or feces (e.g., stray dogs with *Echinococcus*, rodents with *Strongyloides*) could enable non-invasive surveillance of zoonotic parasites.	(86, 87)

“Host miRNA” denotes a microRNA encoded by the animal host's genome (often involved in immune responses), whereas “Parasite/Viral miRNA” indicates a microRNA or analog encoded by the pathogen. Validated means the miRNA's differential expression has been experimentally observed in infected vs. uninfected samples; Predicted refers to in silico or indirect evidence only. All listed miRNAs are proposed as surveillance biomarkers; their detection or level changes in urban wildlife could signal the presence of the corresponding zoonotic pathogen. Citations provide source evidence for each miRNA's relevance.

In summary, miRNAs offer strong potential for early detection of zoonotic infections in urban wildlife due to their stability, broad pathogen coverage, and suitability to minimally invasive sampling. Their practical value lies in the ability to detect subclinical infections before direct pathogen identification is possible, enabling earlier interventions. However, their deployment faces specific limitations, including the need for species-specific reference panels, susceptibility to environmental and physiological confounders, and the cost and technical demands of high-throughput assays. Limited genomic resources for many urban wildlife species also hinder assay standardization across regions. Advancing their role in One Health surveillance will require robust normalization protocols, field validation of multi-pathogen panels, and exploration of portable platforms for routine urban monitoring.

## Critical genes at the host-pathogen interface

A host's genetic makeup can fundamentally shape zoonotic risk, particularly through cellular receptors that pathogens exploit to enter cells. Many emerging pathogens are limited by whether their surface proteins can bind to a given species receptor; even slight sequence differences in these receptor genes can block or permit cross-species transmission (28–30). For example, the ACE2 receptor used by SARS-related coronaviruses varies across wildlife: One survey found the SARS-CoV-2 spike could efficiently use ACE2 orthologs from only 25 of 46 tested bat species. In contrast, the other 21 bat ACE2 variants did not support viral entry (31, 32). Such host-specific ACE2 compatibility explains why only certain bats (or intermediate hosts) can harbor SARS-like viruses. Similarly, the β-coronavirus MERS-CoV uses dipeptidyl peptidase 4 (DPP4) as its entry receptor; notably, both human and bat DPP4 can mediate MERS-CoV entry (33). Consistent with this, adaptive evolution has left signatures on these receptor genes; bat DPP4 exhibits positively selected amino-acid changes at the viral binding interface, and bat ACE2 genes also bear marks of past arms races with SARS-like viruses (33). In rodents, analogous patterns are seen: Viruses such as arenaviruses must adapt to species-specific changes in their host receptor (transferrin receptor 1), which often differ at key binding residues between reservoir rodents and humans (28, 30). In essence, virus-facing host genes (receptors, attachment factors) serve as “critical control points” in spillover; their sequence diversity and evolutionary adaptations determine which pathogens can attach to and infect, thereby delineating the spectrum of zoonotic hosts.

Beyond entry receptors, host immunogenetic factors play an equally critical role in either permitting or curtailing zoonotic pathogens. Genes underpinning the immune interface, from innate pattern-recognition receptors to adaptive antigen-presenting molecules, vary significantly between species and populations, affecting pathogen susceptibility and tolerance. For instance, the highly polymorphic Major Histocompatibility Complex (MHC), which governs antigen presentation, can serve as a key zoonotic risk modifier. Urban wildlife populations sometimes show altered immune gene diversity: Urban ordinary hamsters were found to possess significantly more MHC class II alleles (19 alleles) than their rural counterparts (11 alleles), suggesting that city-dwelling hamsters experience strong pathogen-driven selection for MHC diversity (34). Notably, only ~20% of alleles were shared between urban and rural hamsters, reflecting a divergence in immune gene pools (34). Such diversity could broaden immune recognition of novel pathogens, potentially increasing tolerance to endemic microbes. Meanwhile, innate immune genes often evolve rapidly under pathogen pressure. Toll-like receptors (TLRs), which detect conserved microbial motifs, illustrate this: Bat TLR9, for example, shows evidence of positive selection in its ligand-binding domain, presumably adapting to recognize diverse viral DNA. More striking are the unique immunogenetic adaptations in notorious reservoir hosts like bats. Bats have lost entire gene families involved in inflammasome activation (e.g., the PYHIN DNA sensors) and exhibit dampened inflammatory signaling, while conversely upregulating interferon pathways; certain interferon-regulatory factors and interferon-α are constitutively expressed (34). These changes, occurring in genes critical to immune activation, enable bats to tolerate high viral loads with minimal pathology. Such tolerance-oriented immune gene profiles can turn a species into a long-term pathogen reservoir that sheds viruses while rarely succumbing to them.

Identifying and monitoring these critical host genes in urban fauna can provide valuable biomarkers for zoonotic risk. Hosts carrying pathogen-compatible receptor variants or suboptimal immune genes (e.g., low MHC diversity or dampened inflammasome responses) may be more likely to harbor and transmit zoonotic agents. Conversely, evidence of positive selection in a population's immune genes can indicate historical exposure to specific pathogens, highlighting pathogens to which the animals have adapted. In practice, screening urban wildlife for key receptor variants (such as ACE2 or DPP4 sequences closely resembling human-compatible forms) or for immunogenetic traits linked to pathogen tolerance could help pinpoint high-risk interface areas (35, 36). In brief, the interface genes from cell-surface receptors to immune response elements serve not only as mechanistic drivers of cross-species infection but also as measurable biomarkers of a species' propensity to act as a zoonotic disease reservoir or spillover source. By integrating such genetic indicators into surveillance, we can more quantitatively assess and potentially predict zoonotic outbreak risks in urban environments.

The critical host genes summarized in Table 2 highlight genetic biomarkers at the wildlife–pathogen interface that significantly influence susceptibility and adaptive responses to zoonotic pathogens in urban wildlife populations. Receptor genes such as ACE2 and DPP4 exhibit species-specific polymorphisms that quantitatively affect viral binding affinity, thereby influencing host susceptibility to coronaviruses, including SARS-CoV-2 and MERS-CoV (32, 33). Immunogenetic loci, particularly MHC class II DRB alleles, display heightened allelic diversity and pronounced signatures of positive selection in urban-adapted rodents and bats, reflecting evolutionary adaptation to elevated pathogen pressure (2, 34). Additionally, innate immunity genes, exemplified by TLR9, show clear selection signatures indicative of long-term viral exposure and immune modulation. By integrating quantitative affinity metrics, host–pathogen polymorphisms, and molecular evidence of adaptive evolution from original research studies, these genomic markers collectively offer predictive insights critical for the proactive identification of zoonotic spillover risks, thereby informing targeted surveillance and intervention strategies in urban ecosystems.

**Table 2:** Critical host genes at the wildlife–pathogen interface emerging as surveillance biomarkers for zoonotic diseases in urban wildlife reservoirs.

**Host gene**	**Wildlife host species**	**Pathogen type/ disease**	**Zoonotic status**	**Biomarker type**	**Quantitative data/ polymorphism**	**Sample type**	**Comments**	**References**
ACE2	*Rhinolophus affinis* (horseshoe bats—urban and rural)	SARS-related coronaviruses (SARS-CoV, SARS-CoV-2)	Viral, zoonotic	*In silico* binding affinity and polymorphism	Binding affinity K_D_ ≈ 0.44 μM for RaTG13 RBD to RaACE2; lower (~2.78 μM) for RmACE2 with SARS-CoV-2 RBD; only ~25 of 46 bat ACE2 orthologs allowed entry into cells.	Tissue-derived RNA/DNA	Strong positive selection at 25 critical binding residues; variation predicts susceptibility across bat species.	(32, 88, 89)
DPP4	Pipistrellus bats (urban foragers), domestic camels	MERS-CoV and related merbecoviruses	Viral, zoonotic	Sequence polymorphism	Positively selected amino-acid changes at binding interface regions in bat DPP4 homologs, enhancing MERS-CoV entry capacity.	Host tissue/DNA	Viral adaptation is evident via ACE2/DPP4 interaction; it is a potential indicator of coronavirus host compatibility.	(33, 90–92)
MHC class II DRB alleles	*Peromyscus leucopus* (urban white-footed mice, NYC parks)	Hantaviruses, *Borrelia burgdorferi* (Lyme)	Bacterial, zoonotic	Allelic diversity/ selection signatures	Urban populations carried ~19 DRB alleles vs. ~11 in rural mice; only ~20% allele overlap; high MHC-II diversity correlates with pathogen exposure adaptation.	DNA from blood/ear notch	This study provides evidence of positive selection in urban rodents, signaling adaptation to heightened zoonotic pathogen pressure.	(34, 93, 94)
MHC class II (*Noctilio albiventris* DRB)	Urban-roosting bulldog bats (Panama)	Blood parasites, viruses	Mixed pathogens	Allelic polymorphism and selection	18 DRB alleles identified; significant non-synonymous substitution pattern indicates positive selection across interface genes tied to pathogen resistance.	DNA/RNA	Candidate locus for tracking wildlife adaptive immune response to spillover pathogens in urban roosting bat colonies.	(95)
TLR9 (toll-like receptor 9)	Several urban bat taxa	DNA viruses (herpes-like, adenoviruses)	Potential zoonotic	Evidence of positive selection	Bat TLR9 shows signatures of positive selection in ligand-binding domains, indicative of adaptive immune modulation to viral exposure.	Genomic DNA	Suggests innate immune gene evolution in response to persistent viral exposure in urban-dwelling bat species.	(96)

In summary, host–pathogen interface genes provide mechanistically grounded and quantifiable markers for assessing spillover risk in urban wildlife, enabling targeted surveillance of populations most likely to harbor and transmit pathogens. Their practical value lies in identifying species or individuals with receptor variants or immune profiles compatible with high-consequence pathogens, thereby informing early interventions at the wildlife–human interface. However, application in urban settings faces limitations, including incomplete genomic resources for many species, the need for species-specific assays to capture key polymorphisms and the challenge of distinguishing adaptive signatures driven by pathogen pressure from those shaped by other environmental factors. Addressing these gaps will require coordinated genomic surveys of urban fauna, coupled with standardized genotyping protocols, to ensure the reliable integration of these markers into predictive multi-omics surveillance frameworks.

## Zoonotic bacteria in urban wildlife microbiomes as spillover indicators

Emerging evidence from 16S rRNA sequencing surveys shows that urban-adapted wildlife harbors a wide array of zoonotic bacteria, often at surprisingly high prevalences. Commensal rodents carry diverse pathogenic taxa. For example, a high-throughput 16S screen of 711 synanthropic rodents across West African villages (analogous to urban settings) detected seven major genera of pathogenic bacteria, including *Borrelia, Bartonella, Mycoplasma, Ehrlichia, Rickettsia, Streptobacillus*, and *Orientia*, present in rodent spleens (37). Site-level infection rates ranged from 0 to 90% of individuals for specific genera, and fully 26% of rodents were co-infected by multiple zoonotic pathogens (37). Likewise, 16S profiling of urban rats in Europe identified 14 potentially zoonotic genera, with pathogenic *Leptospira* spp. and *Bartonella tribocorum* confirmed by PCR (38). Notably, over 65% of wild rats had their kidney microbiome dominated (>50% of reads) by a single genus, most often *Streptococcus, Mycoplasma*, or *Leptospira*, underscoring frequent heavy colonization by these microbes (38). These findings highlight *Leptospira* and *Bartonella* in city rodents as prominent risk markers, given their known roles in the transmisión of leptospirosis and bartonellosis transmission to humans.

Urban birds and bats similarly carry pathogenic bacteria that could signal spillover risk. A recent metabarcoding survey of nine urban wildlife species in Madrid found that all hosts harbored some zoonotic taxa, with birds and bats standing out as key reservoirs (39). Urban birds (e.g., house sparrows, pigeons) showed exceptionally high representation of *Campylobacter* and *Listeria* in their fecal microbiota, containing foodborne pathogens under mandatory surveillance in the EU (39). In one 16S study of feral pigeons in Seoul (144 samples), Campylobacter was detected in 19 samples across 13 city districts, Listeria in seven samples, and *Chlamydia* in three samples (40), indicating routine shedding of enteric pathogens onto urban surfaces. Urban-adapted bats can also be sources of unusual bacteria: The Madrid survey detected *Chlamydia* (likely *C. psittaci*-like) and even *Vibrio cholerae* sequences in bat guano (39), highlighting how 16S screening can reveal *Vibrio* and other unexpected genera circulating in city wildlife. Collectively, these studies consistently underline the presence of *Campylobacter, Salmonella, Listeria, Chlamydia*, and other high-risk genera in the gut communities of urban birds and bats, raising concerns that synanthropic wildlife amplify these pathogens in proximity to humans (39, 40).

Perhaps most importantly, microbiome-based surveys suggest that shifts in urban habitat and diet foster conditions conducive to pathogen spillover by altering wildlife gut ecology. Urban animals often harbor less diverse microbiomes, which can facilitate overgrowth of opportunistic microbes. For instance, American white ibises foraging in city parks exhibited significantly reduced gut bacterial diversity and a higher incidence of *Salmonella enterica* shedding compared to wetland conspecifics (41–43). Ibises with the lowest gut diversity were the most likely to test positive for *Salmonella* (44), and birds from highly urbanized sites showed *Salmonella* prevalence on the order of ~25%, with nearly half of the isolates genotypically matching strains from human clinical cases (44). Other urban avifauna show analogous patterns: Urban house sparrows and gulls have less diverse gut microbiomes than rural populations and correspondingly higher frequencies of pathogens like *Yersinia* and *Salmonella* (44). Even urban carnivorous scavengers can serve as sentinels; for example, jungle crows feeding on anthropogenic waste in cities were found to harbor a “relatively high prevalence of potentially pathogenic organisms” in their intestinal flora (45). Together, these studies demonstrate that 16S rRNA metabarcoding is a powerful early-warning tool for detecting zoonotic bacteria in urban wildlife. By quantitatively profiling high-risk genera from *Leptospira* in rats to *Campylobacter* in pigeons, researchers can monitor pathogen abundance in wildlife reservoirs and identify emerging spillover threats before human outbreaks occur (38, 39). This microbiome-based surveillance offers a proactive approach to pinpoint hotspots of zoonotic transmission risk in urban environments and to guide targeted public health interventions.

Complementing these community-level findings, targeted PCR and RT-PCR assays have identified critical bacterial and parasitic pathogens across diverse urban wildlife reservoirs, as detailed in Table 3. Key findings indicate that urban rodents frequently harbor pathogens such as *Bartonella* spp. (67.5% prevalence in urban rats); *Leptospira* spp. (up to 45% in some rodent populations) and *Seoul orthohantavirus* (15%−30% prevalence), emphasizing rodents as pivotal reservoirs of zoonotic diseases. Stray and free-roaming dogs similarly emerge as significant hosts, carrying pathogens like *Leishmania infantum* (~25% prevalence) and *Ehrlichia canis* (~17% PCR-positive, with seroprevalence around 85%), indicating widespread exposure within urban canine populations. Moreover, feral birds, particularly pigeons, present a notable public health risk, shedding pathogens such as *Chlamydia psittaci* (up to 90% prevalence in particular flocks) and *Salmonella* spp. (up to ~33% prevalence) (46–51). Collectively, these data underscore the importance of targeted molecular surveillance to proactively identify zoonotic threats within urban wildlife populations, facilitating timely public health interventions.

**Table 3:** Bacterial and parasitic pathogens detected in urban wildlife reservoirs: prevalence, detection methods and zoonotic implications.

**Pathogen (agent)**	**Main urban host species**	**Detection method**	**Prevalence/notable findings**	**References**
*Bartonella* spp. (rodent-borne)	Norway rats *(Rattus norvegicus*)	PCR (e.g., *gltA* gene)	Very common in urban rats; e.g., 67.5% of tested rats in Los Angeles were PCR-positive for *Bartonella* DNA. Many sequences corresponded to human-pathogenic species (e.g., *B. tribocorum, B*. *elizabethae*).	(50)
*Bartonella henselae* (cat-scratch fever bacterium)	Feral/stray cats (*Ctenocephalides fleas* are vectors)	PCR (e.g., *nuoG* gene)	Detected in roughly 10%−20% of cats in cities. Globally, ~15.3% of cats carry *B. henselae* DNA on average (higher in warm climates and dense stray populations).	(51)
*Chlamydia psittaci* (avian chlamydiosis bacterium)	Feral pigeons (*Columba livia*)	PCR (e.g., 16S/23S rRNA or *ompA* gene)	Present in many urban pigeon flocks. For example, ~10.7% of feral pigeons in one survey were PCR-positive. Reported prevalence varies widely from ~1 to 5% up to >90% in different flocks and methods. pl, indicating pigeons as a notable zoonotic reservoir.	(46)
*Ehrlichia canis* (canine monocytic *ehrlichiosis and rickettsia*)	Stray and free-roaming dogs	PCR (16S rRNA gene sequencing)	Common in tick-exposed dogs. PCR surveys find ~17% of stray dogs positive for *E. canis* DNA. Seroprevalence is often much higher (e.g., ~85% of stray/shelter dogs had *E. canis* antibodies in one study), parasites and vectors, indicating widespread exposure in urban dog populations.	(47)
*Leishmania infantum* (visceral leishmaniasis protozoan)	Stray dogs (urban and peri-urban areas)	PCR (kinetoplast DNA, etc.)	Endemic in certain cities (Mediterranean, Latin America). For example, ~24%−25% of dogs in an endemic region tested PCR-positive for *L. infantum*. Global canine infection prevalence is around 15% in endemic zones, making dogs an important urban reservoir.	(48)
*Leptospira* spp. (pathogenic serovars causing leptospirosis)	Rodents and other mammals (rats, mice; also, raccoons, skunks, opossums, dogs)	PCR (e.g., 16S or *lipL32* gene)	Widely found in city wildlife. In Los Angeles, active infection was detected by PCR in all major species except squirrels, e.g., 15% of skunks, 8.6% of raccoons, ~3%−4% of coyotes were positive. Urban rats often carry *Leptospira* in kidneys, a Madagascar study found 20.2% of rodents positive (with up to ~45% in brown rats). These data underscore the zoonotic risk in urban environments.	
Rabies *Lyssavirus* (rabies virus)	Bats (various urban-adapted bat species); also, feral dogs in some regions	RT-PCR (viral RNA detection)	Typically low prevalence in wild animals, but critical for public health. In urban bat populations, only a few percent are rabid at any time, e.g., about 6.0% average positivity in bats tested in one study (range ~2%−12% across species). Stray dogs in endemic countries can also carry rabies (controlled via vaccination in many cities).	(97)
*Rickettsia felis* (“flea-borne spotted fever” *Rickettsia*)	Cat fleas (*Ctenocephalides felis*) on cats and dogs	PCR (e.g., *gltA* or *ompB* genes)	Very frequent in cat flea populations. For instance, ~25%−30% of cat fleas in a Sicilian city carried *R.felis* DNA. In that survey, nearly half of pets (48% of cats and 46% of dogs) had at least one flea infected with *R. felis*, highlighting the high risk of this zoonotic pathogen in urban homes.	(49)
*Rickettsia rickettsii* (rocky mountain spotted fever agent)	Brown dog ticks (*Rhipicephalus sanguineus*) parasitizing urban dogs	PCR (e.g., *ompA* gene sequencing)	Generally, low infection rates in tick vectors, but significant during outbreaks. In a city epidemic in Mexicali, Mexico, only ~0.7% of ticks were PCR-positive for *R. rickettsii* overall, though hotspot neighborhoods reached ~6% tick infection. Many dogs (up to 65%) showed exposure by serology, emphasizing the need for tick control in urban dog populations.	(98)
*Rickettsia typhi* (murine typhus *Rickettsia*)	Oriental rat fleas (*Xenopsylla cheopis*) on city rodents (rats, opossums)	PCR (e.g., 17-kDa or *gltA* gene)	Established in commensal rodent flea cycles. For example, 24% of rodent fleas in an urban Madagascar study carried *R. typhi* DNA. This flea-borne pathogen causes murine typhus in humans; its presence in city rats and opossums (especially in warm climates) is a known public health concern.	(99)
*Salmonella* spp. (enteric bacteria, e.g., *S. Enteritidis, S. Typhimurium*)	Feral pigeons (also other urban birds and rodents)	PCR or culture (e.g., *invA* gene PCR, stool culture)	Urban birds often shed *Salmonella*. Surveys in Europe show ~5%−10% of feral pigeons on average carry *Salmonella*. In Brussels, a high prevalence of ~33% *S. Enteritidis* was reported in pigeons. Such birds can contaminate public spaces and potentially transmit zoonotic salmonellosis.	(100)
Seoul orthohantavirus (Seoul virus, a rodent-borne hantavirus)	Brown rats (*Rattus norvegicus*)	RT-PCR (viral RNA in tissues)	Enzootic in urban rat populations worldwide. Infection rates of ~15%−30% have been recorded in wild rats in some cities (e.g., Seoul, South Korea). Seoul virus can cause hemorrhagic fever with renal syndrome in humans; its presence in city rats (often asymptomatic carriers) makes it an important One Health biomarker in urban settings.	(101)
*Toxoplasma gondii* (toxoplasmosis protozoan)	Feral cats (definitive hosts shedding oocysts); also, urban birds and rodents (intermediate hosts)	PCR (e.g., *B1* gene, ITS region)	Feral cats commonly carry and spread Toxoplasma. In one study from Seoul, ~30.6% of stray cats were PCR-positive for *T. gondii* DNA (vs. 0% of pet cats). Similarly, a California survey found ~25.9% of feral cat fecal samples positive for *T. gondii* oocyst DNA. These high rates highlight stray cats as a key source of environmental oocyst contamination in cities.	(102)
*Trypanosoma cruzi* (Chagas disease parasite)	Opossums and raccoons (urban wild mammals); also stray dogs and cats to lesser extent	PCR (kinetoplast/ minicircle DNA)	Detected in certain urban wildlife reservoirs. For example, in a U.S. city focus, ~50.9% of Virginia opossums and 42.2% of raccoons were PCR-positive for *T. cruzi*, indicating active infections. Stray dogs and cats can also be infected. These findings demonstrate that the Chagas parasite circulates even in urban environments, maintained by wild mammals.	(103)

All data above are from recent studies and surveys. Prevalence values are illustrative (exact rates vary by city and research). Detection methods listed indicate how each pathogen was identified (e.g., broad range 16S rRNA gene sequencing vs. specific PCR assays). Each biomarker highlighted can serve as a target for screening urban wildlife and feral animal samples for zoonotic disease surveillance. Citations correspond to source studies for the provided data.

In summary, microbiome profiling via 16S rRNA and targeted molecular assays offers a sensitive means to detect high-risk bacterial taxa in urban wildlife, enabling early identification of spillover threats and mapping of transmission hotspots. Its practical strength lies in capturing both known and unexpected pathogens across multiple host species, providing a broad surveillance net. However, its application in urban contexts is limited by the need for standardized analytical pipelines, the challenge of distinguishing transient environmental contamination from actual colonization, and gaps in understanding how microbiome shifts relate to pathogen shedding and transmission risk. Integrating longitudinal sampling with harmonized bioinformatic workflows will be essential to translate these microbial indicators into actionable components of multi-omics urban surveillance frameworks.

## Viromics (viral metagenomics): unveiling zoonotic “dark matter”

Viral metagenomics (or *viromics*), is an unbiased culture-independent approach that allows simultaneous detection of known viruses and novel, previously undescribed viruses in wildlife samples (52, 53). By sequencing all viral genetic material present, this method reveals the vast “viral dark matter,” the multitude of viral sequences with no close relatives that traditional diagnostic methods would miss (52–54). Viromics thus enables early surveillance of emerging pathogens without requiring prior cultivation or targeted PCR assays, making it invaluable for proactive zoonotic disease monitoring (54). Given that many recent outbreaks (e.g., SARS, MERS, Ebola) originated from animal hosts, metagenomic profiling of wildlife viromes can provide critical forewarning by identifying novel viruses directly in their natural reservoirs.

Key examples demonstrate viromics' power to uncover hidden zoonoses. In bats, metagenomic surveys have revealed numerous novel coronaviruses and other viruses, while also detecting pathogens closely related to known human viruses (55). These findings confirmed bats as reservoirs for diverse coronaviruses (including SARS-related viruses) long before some of these pathogens spilled over into humans (55). Likewise, in rodents, viromics has revealed unexpected zoonotic agents; for instance, a recent study of wild rodents in Brazil detected viral contigs from hemorrhagic fever virus families (*Filoviridae* and *Arenaviridae*) (56), underscoring that even standard mice and rats may harbor Ebola- or Lassa-like viruses. Non-human primates have also been shown to carry viruses with human pandemic potential. A viral metagenomic screen identified a human-like astrovirus in chimpanzee feces (57), highlighting the risk of cross-species transmission among our closest relatives.

Applying viromics to urban wildlife is a promising yet underexplored frontier (58). Synanthropic species (city-dwelling animals such as rodents, bats, and pigeons) live near humans, making the characterization of their viromes highly relevant for public health. Although initial data are limited, they already indicate that urban fauna harbor a heterogeneous mix of pathogens and novel viruses. For example, a viromic analysis of wild Norway rats from an inner-city environment revealed a variety of known enteric viruses (e.g., rotaviruses and noroviruses) alongside entirely new viruses showing very low similarity to any known strains (59). Such findings illustrate how viromics can guide researchers in identifying key viral biomarkers of zoonotic risk even in urban settings. By profiling the whole viral community in these animals, one can pinpoint candidate viral indicators (from familiar agents to cryptic new viruses) that deserve monitoring, an approach that is essential to illuminate the urban virome “dark matter” and to bolster early-warning systems for emerging diseases (58, 59).

In line with this approach, Table 4 summarizes critical viral taxa detected through untargeted viral metagenomic approaches and confirmatory PCR in urban and peri-urban wildlife, highlighting their zoonotic relevance and potential as early-warning biomarkers for zoonotic transmission. Notably, Seoul orthohantavirus, a confirmed zoonotic pathogen causing hemorrhagic fever with renal syndrome, was identified in urban brown rats from Liverpool via unbiased RNA metagenomics, yielding a substantial 4.8 kb genomic contig. Similarly, rat hepatitis E virus, a suspected zoonotic agent implicated in sporadic human hepatitis cases, was detected at high prevalence in peri-urban rodents in Yunnan, China (60, 61). Novel viruses of unknown pathogenicity, such as Beilong and Tailam paramyxoviruses, in urban rodent populations and newly characterized bat-associated betacoronaviruses and sapoviruses, highlight the broad utility of viromics in revealing previously undetected viral diversity (62). Collectively, these findings demonstrate viromics' capacity to uncover both known zoonotic threats and cryptic viral taxa circulating in wildlife and provide critical biomarkers for proactive zoonotic surveillance in urban ecosystems.

**Table 4:** Viruses identified via viromics and confirmatory PCR in urban and peri-urban wildlife: host species, geographic location, zoonotic relevance, and quantitative findings.

**Virus (taxonomy)**	**Host species**	**Location**	**Detection method**	**Sample type**	**Zoonotic relevance**	**Quantitative data**	**Reference**
Seoul orthohantavirus (*Orthohantavirus, Hantaviridae*)	Brown rat (*Rattus norvegicus*)	Liverpool, UK (urban)	Untargeted RNA metagenomic sequencing; partial viral genome (~4.8 kb contig) assembled	Lung and intestinal tissue (pooled)	Confirmed zoonotic, causes hemorrhagic fever with renal syndrome in humans.	Detected in one out of dozens of rats; the largest contig ~4.8 kb confirmed as Seoul virus.	(60, 61, 104)
Oxbow orthohantavirus (*Orthohantavirus, Hantaviridae*)	Brown rat (*R. norvegicus*) and wood mouse (*Apodemus sylvaticus*)	Liverpool, UK (urban)	Untargeted RNA metagenomic sequencing (NGS)	Lung, gut tissues, and feces (pooled)	Unknown, no human cases; frequently detected in rodents (first identified in the shrew mole).	Present in 16 rat samples and six mouse samples (multiple individuals/pools) by NGS.	(104)
Rat hepatitis E virus (*Orthohepevirus* C, *Hepeviridae*)	Brown rat (*R. norvegicus*) and Asian house rat (*R. flavipectus*)	Yunnan, China (peri-urban)	Viral metagenomic sequencing with RT-PCR confirmation	Mixed organ tissues (e.g., liver, intestine)	Suspected zoonotic rat HEV has caused sporadic hepatitis E in humans (immunosuppressed cases).	Detected in multiple rodents; widely distributed in surveyed rat populations (dominant in this region's virome).	(62, 105)
Beilong virus (*Paramyxoviridae*, genus *Jeilongvirus*)	Brown rat (*R. norvegicus*)	Heilongjiang, China (peri-urban)	Viral metagenomic sequencing (NGS) with specific RT-PCR validation	Mixed organ tissues (pooled from rats)	Unknown, no known human infections (rodent *Jeilongvirus* group).	Detected in two of eight pooled samples (from five rats each); among the most abundant viral taxa in rats.	(62)
Tailam virus (*Paramyxoviridae*, genus *Jeilongvirus*)	Asian house rat (*R. flavipectus*)	Yunnan, China (urban)	Viral metagenomic sequencing (NGS) with RT-PCR confirmation	Mixed organ tissues (pooled from rats)	Unknown, no known human infections (rodent *Jeilongvirus* group).	Detected in rats from Yunnan; closely related to Beilong virus (forming a distinct rat paramyxovirus clade)	(62)
Novel bat betacoronavirus (*Nobecovirus* subgenus, Coronaviridae)	Gray-headed flying fox (*Pteropus poliocephalus*)	Eastern Australia (urban/ suburban colonies)	Unbiased metatranscriptomic virome sequencing (RNA-seq)	Fecal (bat guano) samples	Unknown, bat-specific lineage (no human cases to date).	Detected in two colonies ~1,375 km apart, indicating widespread circulation in urban bat roosts	(106)
Bat sapovirus (*Caliciviridae*; novel strain)	Gray-headed flying fox (*P. poliocephalus*)	Eastern Australia (urban/suburban)	Unbiased metatranscriptomic sequencing (RNA virome analysis)	Fecal (guano) samples	Unknown, first bat sapovirus identified (pathogenicity undetermined).	First identification of a bat sapovirus in Australian bats; detected in one urban colony (index of discovery)	(106)
Bat astrovirus (Mamastrovirus, *Astroviridae*), frequently detected	Insectivorous bats (e.g., Myotis, *Rhinolophus* spp.)	Multiple locales (e.g., Asia and Europe; urban-adjacent roosts)	Viral metagenomic surveys of bat fecal viromes across sites	Fecal samples (bat guano)	Unknown, no confirmed human infections; commonly found in bat viromes	High prevalence and genetic diversity in bat populations (astroviruses detected in bats with significant frequency)	(107)
West Nile virus (Flavivirus, *Flaviviridae*)	Wild urban birds (e.g., American crow; *Corvus brachyrhynchos*)	New York City, USA (urban)	Unbiased viral genome sequencing of bird tissues (discovery during unexplained die-off)	Brain and organ tissues of dead birds	Confirmed zoonotic—causes West Nile fever/encephalitis in humans	Thousands of urban birds infected during 1999–2000 outbreak (e.g., 67% of tested crows positive; >3,400 dead birds tested)	(2, 108)

In summary, viromics offers an unparalleled capacity to detect both known and novel viral agents in urban wildlife, providing early-warning insights into potential spillover threats before they are clinically recognized. Its unbiased nature allows simultaneous surveillance of multiple viral families, making it particularly valuable in complex urban ecosystems where pathogen diversity is high. However, its routine application is constrained by high costs, intensive bioinformatic requirements, and challenges in interpreting the pathogenic significance of novel or low-similarity viral sequences. Standardizing analytical pipelines, expanding reference databases, and integrating viromic outputs with ecological and epidemiological data will be essential to translate these discoveries into actionable components of predictive multi-omics surveillance.

## Host transcriptomics (RNA-seq) in response to infections

Wild animals that serve as disease reservoirs often show few clinical signs when infected, making it challenging to assess their immune status. In the context of urban wild fauna and zoonoses, global transcriptomic analysis (RNA-seq) of the host's response is invaluable. By capturing the expression of thousands of genes simultaneously, researchers can detect subtle molecular changes that occur even in asymptomatic infections (63). This approach provides a comprehensive overview of the host's early immune reaction, helping to identify potential biomarkers of disease in non-model species. In other words, RNA-seq enables monitoring of immune responses in wild populations and pinpointing gene expression patterns that signal an infection, which is crucial for early warning and surveillance of zoonotic outbreaks (63).

A key advantage of whole-transcriptome profiling is its ability to uncover central regulatory genes and altered pathways during the initial phases of infection. Unlike targeted assays, RNA-seq is unbiased and can reveal which host genes are activated or repressed in response to a pathogen. This enables researchers to identify master regulators (for example, interferon-related transcription factors) as well as disrupted metabolic and signaling pathways. Studies have shown that early infections often trigger a strong interferon-mediated immune response while simultaneously affecting metabolic processes (64). For instance, Zika virus infections have been found to stimulate interferon production and concurrently downregulate metabolism-related pathways in host cells (64). Likewise, transcriptomic data can highlight genes such as cytokines, pattern-recognition receptors, and other immune modulators that act as hubs within the response network (65). Identifying these central nodes is critical, as they can indicate how the host mobilizes its defenses and which pathways are most perturbed. Such insights not only deepen our understanding of host-pathogen interactions but also point to candidate genes that could serve as early molecular biomarkers of infection or targets for intervention. Table 5 compiles experimentally validated and statistically significant differentially expressed genes (DEGs) identified through RNA-seq studies in urban and peri-urban wild fauna infected with zoonotic pathogens. It details both upregulated and downregulated transcripts, their associated immune or metabolic pathways, host species, and the specific pathogens involved. Notably, innate immune regulators such as *IL-6, TNF-*α, and *IFIT3* show strong and consistent upregulation across multiple taxa and infection types, underscoring their utility as cross-species biomarkers of early pathogen exposure. Conversely, the downregulation of anti-apoptotic genes like *BCL2* during severe viral infections highlights pathway-specific vulnerabilities that could be exploited for diagnostic purposes. By pairing gene-level changes with pathway context and quantitative metrics, this table offers a practical reference for researchers aiming to identify and validate transcriptomic biomarkers of zoonotic infection in field-collected wildlife samples.

**Table 5:** Differentially expressed genes (DEGs) in urban/peri-urban wild fauna hosts in response to zoonotic infections.

**Regulation**	**Immune/ metabolic pathway**	**Wildlife host (prban/peri-urban)**	**Pathogen (disease)**	**Study type**	**Tissue/ sample**	**Quantitative change**	**Reference**
IL-6 (upregulated)	Pro-inflammatory cytokine (acute-phase response; JAK–STAT pathway)	Black rat (*Rattus rattus*; urban wild)	*Leptospira* spp. (leptospirosis)	Field (natural infection)	Liver (early infection stage)	Expressed early in infection, significant upregulation in the liver during the initial stage (vs. uninfected controls)	(109)
TNF-α (upregulated)	Pro-inflammatory cytokine (TNF/NF-κB signaling)	Black rat (*Rattus rattus*; urban wild)	*Leptospira* spp. (leptospirosis)	Field (natural infection)	Kidney (late infection stage)	Strongly upregulated in late-stage infection, higher transcript levels in the kidney during advanced disease (adj. *p* < 0.05)	(109)
IL-10 (upregulated)	Anti-inflammatory cytokine (regulatory immune response)	Black rat (*Rattus rattus*; urban wild)	*Leptospira* spp. (leptospirosis)	Field (natural infection)	Kidney (late infection stage)	Elevated expression in late infection, increased IL-10 transcripts in kidneys during the chronic phase (modulating inflammation)	(109)
IFIT3 (upregulated)	Interferon-stimulated gene (innate antiviral RIG-I-like receptor pathway)	Jamaican fruit bat (*Artibeus jamaicensis*; peri-urban)	Tacaribe virus (New World arenavirus; hemorrhagic fever model)	Experimental (viral challenge)	Multiple (spleen, liver, kidney)	Significantly upregulated in infected bats high IFIT3 mRNA induction in ≥2 organs (robust type-I IFN response; FDR < 0.05)	(110)
SOCS1 (upregulated)	Suppressor of cytokine signaling (negative feedback in JAK–STAT pathway)	Jamaican fruit bat (*Artibeus jamaicensis*)	Tacaribe virus (New World arenavirus)	Experimental (viral challenge)	Multiple (spleen, liver, kidney)	Markedly upregulated increased SOCS1 transcripts in infected bats (in ≥2 tissues; FDR < 0.05), indicating activation of cytokine-negative regulators	(110)
BCL2 (downregulated)	Anti-apoptotic regulator (intrinsic apoptosis pathway)	Jamaican fruit bat (*Artibeus jamaicensis*)	Tacaribe virus (fatal hemorrhagic disease in bats)	Experimental (viral challenge)	Spleen and Liver (late infection)	Downregulated in spleen and liver—significantly lower BCL-2 mRNA vs. controls (correlated with enhanced apoptosis in those organs; *p* < 0.05)	(110)
DDX58/RIG-I (upregulated)	Viral RNA sensor (RIG-I–like receptor initiating interferon responses)	Zebra finch (*Taeniopygia guttata*; model passerine)	West Nile virus (WNV encephalitis)	Experimental (lab infection)	Spleen (peak viremia, day 4 p.i.)	Upregulated during acute infection part of a suite of ≥5 RLR-pathway genes induced (average log_2_FC ≈ 1.5–2.0, FDR < 0.05)	(110)
IL-4 (upregulated)	T-helper 2 (Th2) cytokine (B-cell help and antibody class switching)	Deer mouse (*Peromyscus maniculatus*; peri-domestic rodent)	Andes virus (Hantavirus, HCPS)	Experimental (viral challenge)	Lymph nodes (stimulated T cells)	Robust upregulation IL-4 pathway genes strongly induced in infected deer mice (prominent Th2/Tfh response, e.g., elevated IL-4 mRNA in antigen-stimulated cells, *p* < 0.01)	(24)

Bats are a prime example of wild (often urban-adapted) animals where transcriptomics has illuminated the host response to infection. Bats are notorious reservoirs for coronaviruses, henipaviruses (like Nipah), and other zoonotic viruses, yet they typically tolerate these pathogens with minimal illness (65). Transcriptome-wide analyses have helped explain this resilience. For instance, a study on fruit bats (genus *Pteropus*) used RNA-seq to profile bat cells after viral infection, revealing a robust induction of antiviral genes (65). Over 200 genes were differentially expressed, with type I interferon (IFN-β) and many interferon-stimulated genes (ISGs) highly upregulated (e.g., RIG-I, MDA5, ISG15, IRF1). Notably, the bat cells also upregulated genes not previously known to be antiviral, such as *MORC3*, highlighting novel components of bat immunity. This broad innate response was observed when bat cells were challenged with a strong immune stimulus (an RNA virus), but the Nipah virus could actively suppress the response. When the same bat cells were infected with Nipah (which carries interferon-antagonist proteins), those key immune genes failed to be induced. Introducing Nipah's antagonist proteins into a test virus significantly attenuated the bat's gene expression response (65). This transcriptomic evidence shows how the Nipah virus undermines the bat's antiviral gene networks, and it underscores the power of RNA-seq to detect such host-pathogen dynamics. In the case of bat coronaviruses, transcriptomic studies have revealed equally intriguing adaptations. One recent analysis found that bat cells responding to a SARS coronavirus (SARS-CoV-2) trigger alternative antiviral pathways: key interferon-stimulated genes like OAS1 and MX1 were strongly upregulated, while the typical PKR pathway remained muted (66). This suggests bats rely on distinct RNA-processing mechanisms (e.g., Dicer-mediated viral RNA degradation) and a controlled inflammatory response to tolerate coronavirus infection. In summary, global gene expression profiling in bats has identified central immune regulators (IFNs, IRFs) and unique pathway shifts that allow these urban-associated mammals to harbor lethal viruses without succumbing, information that is invaluable for understanding viral tolerance and identifying genetic markers of infection in bat populations.

Non-human primates provide another compelling example, especially for zoonotic viruses like Zika. Many primate species (e.g., macaques) live in or around human settlements, making them relevant “urban wild fauna” in zoonotic disease cycles. Transcriptomic studies in primates infected with Zika virus (ZIKV) have shed light on the host's early response and potential biomarkers. In one study, researchers infected rhesus and cynomolgus macaques with ZIKV and performed blood transcriptome profiling to capture the acute phase response (67). Within just a few days post-infection, the monkeys showed a sharp upregulation of innate immune genes in peripheral blood. Specifically, there was heightened expression of pattern-recognition receptors like RIG-I (*DDX58*) and MDA5 (*IFIH1*), several Toll-like receptors, and key interferon-regulatory factors (such as IRF7). Correspondingly, a suite of interferon-stimulated genes was strongly induced, including IFIT1/2/3, MX2, OAS2, and OAS3, among others, indicating that a systemic antiviral state was rapidly established (67). This global transcriptomic activation aligns with the observed immune outcomes in these primates, which developed robust antiviral defenses (e.g., producing neutralizing antibodies and T-cell responses) and successfully controlled the infection (67). The ability of RNA-seq to capture such comprehensive immune signatures is crucial. In the Zika-infected primates, it not only confirmed the central role of the interferon pathway in acute infection but also provided molecular readouts (gene expression signatures) that could serve as indicators of infection in wild primate populations. For example, an elevated transcript level of genes like *ISG15* or *MX2* in a free-ranging monkey might be a telltale sign of flavivirus exposure. Moreover, transcriptomic comparisons can reveal differences between species or conditions. In the Zika study, specific gene expression patterns differed depending on the presence or absence of specific immune cells (67), offering insight into how various components of the immune system contribute to fighting the virus.

In summary, host transcriptomics (RNA-seq) is a powerful tool for dissecting the infection response in urban wild fauna species. It provides a global, unbiased view that is especially useful for non-model organisms like bats, birds, or primates, where prior knowledge of immune markers may be limited. By surveying the entire host genome's response, researchers can identify key regulatory genes (e.g., interferons, transcription factors) and altered pathways (immune signaling cascades, metabolic shifts) that characterize the early stage of infection (64, 65). These insights deepen our understanding of why reservoir hosts often tolerate pathogens and which host factors are linked to either resistance or disease progression. Practically, transcriptomic profiles also point to potential biomarkers of zoonotic infection. For instance, a consistent upregulation of certain ISGs or inflammatory regulators in infected hosts could be used to develop blood tests or molecular surveillance tools in wild populations (63). As the cost of RNA sequencing falls and genomic tools expand, this kind of holistic gene expression analysis is becoming more feasible in field studies. Ultimately, integrating transcriptomics into wildlife disease research equips scientists and public health experts with a detailed molecular “readout” of the host's health status, enhancing our ability to detect and respond to emerging zoonoses at their source.

In summary, host transcriptomics offers a comprehensive and unbiased means to detect early immune responses in urban wild fauna, enabling the identification of infection signatures even in asymptomatic carriers. Its practical value lies in revealing pathway-level changes and key regulatory genes that can serve as robust biomarkers across species. However, its application is limited by the need for high-quality RNA from field samples, the complexity of bioinformatic analyses, and the challenge of distinguishing pathogen-specific responses from other stressors. Standardizing sampling protocols, expanding annotated reference genomes, and integrating transcriptomic outputs with other omics layers will be essential to translate these insights into reliable, actionable tools for urban zoonotic surveillance.

## Critical assessment of a multi-omics integration framework for zoonotic surveillance in urban wild fauna

Holistic multi-omics integration is increasingly recognized as essential for understanding complex host–pathogen–microbiome interactions. Traditional single-omic approaches often miss interdependencies between biological layers; in contrast, combining data from multiple “omes” can reveal mechanistic insights that would otherwise remain hidden (68). For example, integrated analysis of the gut microbiota and host transcriptome has demonstrated that commensal microbes can directly influence host gene expression (69). The proposed framework simultaneously profiles five key molecular levels: (i) host microRNAs (specific early biomarkers of infection), (ii) host–pathogen genetic factors (e.g., immune receptor polymorphisms and other immunogenetic markers), (iii) the bacterial microbiota composition (16S rRNA profiling), (iv) the virome (viral metagenomic analysis), and (v) the host's global transcriptome (capturing functional responses). Each component contributes unique information. Notably, microRNAs are stable, readily detectable indicators of early physiological responses to infection (70), while parallel sequencing of host and pathogen genomes can pinpoint genetic variants associated with host resistance or pathogen virulence (71, 72). By interrogating all these layers in tandem, this integrative approach promises a comprehensive, systems-level view of infection dynamics and host responses.

A range of integrative strategies and analytical frameworks are now emerging to handle such multimodal data. One approach is to apply unified sequencing techniques that capture multiple omics from the same specimen. For instance, a single optimized metatranscriptomic assay has been shown to concurrently profile the respiratory RNA virome, the bacterial microbiome, and the host transcriptomic response directly from a low-input nasal swab (73). This method successfully sequenced RNA viruses and identified commensal and pathogenic bacteria while simultaneously measuring the host's immune gene expression (73). Notably, it uncovered unexpected co-infections (e.g., frequent asymptomatic RSV and coronavirus presence in children). It linked microbial activity with upregulation of host antiviral and inflammasome pathways (73), underscoring the power of simultaneous multi-omic surveillance. Beyond such single-assay innovations, integration can also be achieved by combining distinct datasets *post hoc* using advanced computational tools. As more omics layers are incorporated, increasingly sophisticated statistical and machine-learning methods are being employed to uncover correlations and causal links across these datasets (74). Techniques such as canonical correlation analysis, network inference, and multi-dimensional clustering have been applied to disentangle the intricate cross-talk among host genes, microbes, and molecular biomarkers. Ensuring robust integration of these high-dimensional data requires careful normalization and modeling, and this remains an active area of development in systems biology.

Implementing a multi-omic approach in practice faces several challenges. From a methodological standpoint, obtaining all relevant biomolecules from non-invasive samples (e.g., feces, saliva, swabs) can be difficult. Such samples often yield limited and degradation-prone nucleic acids, complicating the concurrent analysis of DNA (for microbiome/virome) and RNA (for transcriptome/miRNAs). Indeed, even with specialized protocols, further optimization of sample preservation and extraction may be needed to recover high-quality RNA and DNA from low-biomass field samples (73). Another hurdle is the sheer volume and complexity of data generated: parallel sequencing of host transcripts, microbial 16S amplicons, viral genomes, and small RNAs demands substantial bioinformatic capacity. In regions with limited resources, the lack of local computational infrastructure and expertise can lead to analytic bottlenecks (75, 76). This often necessitates external support for data processing and analysis, highlighting the need to build regional bioinformatics capacity. Additionally, there is currently no universally accepted pipeline for integrating such disparate data types; each study tends to develop a custom approach, making it hard to compare results across projects. This lack of standardization in multi-omics data integration is a notable limitation acknowledged by experts (74).

Looking ahead, several steps can be taken to address these challenges and fully realize the potential of multi-omic integration. First, the development of standardized protocols and best-practice guidelines is critical. Harmonizing how different omics data are generated, normalized and analyzed will improve reproducibility and allow meaningful cross-comparison between studies (77–80). Equally important is the creation and curation of open-access databases that aggregate multi-omics datasets. Large, publicly available multi-omic repositories would enable researchers to share data, validate findings and uncover broad patterns, thereby accelerating discovery (74). Building collaborative networks and infrastructure is especially pertinent in regions like Latin America. For example, international initiatives such as the CABANA project demonstrate the value of coordinated efforts to strengthen bioinformatics capacity in Latin America, focusing on local challenges like communicable disease (75). We propose establishing regional consortia and data-sharing platforms in Latin America (including Mexico) to support multi-omics research, leveraging collective expertise and resources to overcome individual limitations. Such collaborative, interdisciplinary networks would connect field ecologists and molecular biologists, facilitating the exchange of samples, data, and insights. In summary, by investing in methodological innovations, capacity building and open collaboration. The multi-omic integration strategy can be advanced into a powerful tool for high-resolution, real-time understanding of host–pathogen ecosystems.

## Conclusion

The integration of multi-omics surveillance, encompassing miRNA profiling, host–pathogen interface genotyping, bacterial microbiome characterization, viromics, and host transcriptomics, has the potential to transform how we anticipate, detect, and monitor emerging zoonotic risks. By uniting these complementary molecular layers within a single analytical framework, we can detect infection signals across independent biological systems, validate them in real time, and map their ecological and evolutionary contexts. This convergence enables a shift from reactive pathogen detection toward proactive, predictive surveillance capable of identifying cryptic threats before they manifest as outbreaks.

For megadiverse regions such as Latin America and Southeast Asia, where pathogen richness, host diversity, and rapid environmental change converge, the benefits of this approach are particularly pronounced. These regions often harbor high densities of synanthropic wildlife in close contact with human populations, yet surveillance remains fragmented and biased toward known threats. Multi-omics integration directly addresses these limitations: miRNAs can provide early, host-specific signatures of infection; host gene polymorphisms offer insight into susceptibility landscapes; 16S rRNA profiling reveals shifts toward pathogenic bacterial communities; viromics exposes both recognized and novel viral agents; and transcriptomics captures functional immune responses as they unfold. When combined, these layers produce a multidimensional risk profile that can inform targeted interventions with unprecedented precision.

However, realizing this potential requires addressing practical constraints, ensuring nucleic acid preservation from non-invasive samples in field conditions, expanding regional bioinformatics infrastructure, and establishing standardized, interoperable pipelines. Equally critical is the development of open, regionally inclusive data platforms and collaborative networks to democratize access to high-resolution surveillance outputs. By embedding such an integrated system within a One Health framework, we can link ecological, veterinary, and public health intelligence, enabling interventions that are both evidence-driven and context-specific.

In sum, an integrated multi-omics strategy is not simply an enhancement to existing surveillance; it is a paradigm shift toward anticipatory zoonotic risk management. Its application in biodiversity-rich, high-risk regions offers a pathway to bridge critical knowledge gaps, accelerate outbreak forecasting, and safeguard human and ecosystem health before subsequent spillover occurs.
